# Apoptosis Induction in HepG2 and HCT116 Cells by a Novel Quercetin-Zinc (II) Complex: Enhanced Absorption of Quercetin and Zinc (II)

**DOI:** 10.3390/ijms242417457

**Published:** 2023-12-14

**Authors:** Mizuki Nakamura, Daigo Urakawa, Ziyu He, Isao Akagi, De-Xing Hou, Kozue Sakao

**Affiliations:** 1 Graduate School of Agriculture, Forestry and Fisheries, Kagoshima University, Kagoshima 890-0065, Japan; k6572559@kadai.jp (M.N.); k6044078@kadai.jp (D.U.); akagi@agri.kagoshima-u.ac.jp (I.A.); k8469751@kadai.jp (D.-X.H.); 2 The United Graduate School of Agriculture Sciences, Kagoshima University, Kagoshima 890-0065, Japan; k7715393@kadai.jp

**Keywords:** quercetin–zinc (II) complexes, chelating, anticancer, ionophore

## Abstract

Quercetin forms complexes with various metals due to its structural attributes. It predominantly exhibits chelating activity at the 3-hydroxy/4-carbonyl group. Previously, coordination in synthetically obtained quercetin–zinc (II) complexes has been limited to this group. However, the expanded coordination observed in quercetin–iron complexes has opened avenues for diverse applications. Thus, synthesizing novel quercetin–zinc complexes with different coordination positions is a significant advance. In our study, we not only synthesized and comprehensively characterized a new quercetin–zinc (II) complex, Zn-Q, but also evaluated the structure and bioactivity of chelate complexes (Q+Zn) derived from co-treatment in cell culture mediums. The structure of the new compound Zn-Q was comprehensively characterized using 1D ^1^H and 2D correlation spectroscopy (COSY), nuclear magnetic resonance (NMR), Fourier-transform infrared spectroscopy (FT-IR), ultraviolet–visible spectroscopy (UV-Vis), electrospray ionization mass spectrometer (ESI-MS), and X-ray diffraction analysis (XRD) analysis. Subcellular localization and absorption of these zinc (II) complexes were determined using the ZnAF-2 DA zinc ion fluorescence probe. Throughout the experiments, both Zn-Q and Q+Zn exhibited significant antioxidant, cell growth inhibitory, and anticancer effects in HepG2 and HCT116 cells, with Zn-Q showing the highest potential for inducing apoptosis via the caspase pathway. Tracking intracellular zinc complex absorption using zinc fluorescent probes revealed zinc (II) localization around the cell nucleus. Interestingly, there was a proportional increase in intracellular quercetin absorption in conjunction with zinc (II) uptake. Our research highlights the advantages of quercetin complexation with zinc (II): enhanced anticancer efficacy compared to the parent compound and improved bioavailability of both quercetin and zinc (II). Notably, our findings, which include enhanced intracellular uptake of both quercetin and zinc (II) upon complex formation and its implications in apoptosis, contribute significantly to the understanding of metal–polyphenol complexes. Moving forward, comprehensive functional assessments and insights into its mechanism of action, supported by animal studies, are anticipated.

## 1. Introduction

Quercetin is prominently found in vegetables and fruits and stands out for its robust antioxidant properties among flavonoids. This compound is believed to play a pivotal role in warding off various ailments, including cancer [1], arteriosclerosis [2], cardiovascular [3], and diabetes [4] risk, all of which are linked to the oxidative stress instigated by reactive oxygen species [5].

Numerous studies have highlighted the remarkable effects of quercetin against cancers with significant global incidence rates, such as breast, colon, and liver cancers [6]. In particular, the antioxidant and reactive oxygen species scavenging effects of quercetin due to its chelating activity are points of emphasis. This specific action of quercetin plays a crucial role in inhibiting cancer cell proliferation and metastasis.

Several quercetin metal complexes have been obtained to improve the antioxidant activity of quercetin [7,8]. These complexes exhibit activity against a spectrum of cancer cells, including human liver (HepG2) [9,10], breast (MCF-7) [11], pancreatic (MIA-Pa-Ca-2) [12], bladder (BFCT-905) [13], and prostate (PC-3) [14] cancer cells and is expected to be used as an anticancer agent. Structurally, quercetin possesses three chelating sites apt for metal ion interactions: site I (3-hydroxy/4-carbonyl group) or site II (5-hydroxy/4-carbonyl group) located on the C ring and site III (3′,4′-dihydroxy group) on the B ring. Recent studies suggest that the chelating properties of flavonoids arise from site I or site II rather than from the ortho-hydroxyl group on the B ring [15,16]. Consequently, many synthesized quercetin metal complexes feature metal ions predominantly bonded to site I [17,18,19]. Nevertheless, quercetin–iron complexes have been successfully synthesized with iron coordinated to chelating sites other than to site I. This positions quercetin–iron complexes beyond its traditional anticancer role, showcasing its potential in MRI imaging, enhancing circulating proangiogenic cell differentiation [20] and as a preconditioning agent for ischemic diseases and chronic wounds [21]. In contrast, to the best of our knowledge, in quercetin–metal complexes (quercetin: metal ion = 2:1), the coordination of the zinc ion in the quercetin–zinc (II) complex is observed only at site I. Many quercetin–zinc complexes are obtained by allowing a reaction between zinc salts such as zinc chloride or zinc acetate anhydrous and quercetin in a molar ratio of 1:2 [18,19,22]. The reaction conditions range from room temperature to 80 °C, with stirring times ranging from 1.5 to 10 h. The addition of bases such as sodium ethoxide [23], NaOH [24] or triethylamine [22] has also been reported. Under all reaction conditions, the zinc ion binds mainly to the 3′,4′-dihydroxy group of the B ring of quercetin or forms a complex in which the 3-hydroxy and 4-carbonyl groups of two quercetin molecules sandwich the zinc ion [13,25]. In addition, the reported quercetin–zinc (II) complexes exhibit higher biological activity than the parent compound. However, the detailed mechanism, especially concerning anticancer activity, remains elusive. Furthermore, despite the various experimental studies that have been conducted to date, very few reports have focused on the metal chelation of quercetin in solution, especially in an aqueous biological environment, and on the associated changes in molecular structure and on the biochemical effects.

In this study, we synthesized a quercetin–zinc (II) complex (Zn-Q) characterized by a novel structural feature wherein zinc (II) is coordinated to the 3′,4′-dihydroxy group (Site III). We subsequently assessed the anticancer activity of these synthesized complexes. In the evaluation of cultured cells, we also examined the chelate structure formed in a culture medium through co-treatment with quercetin and zinc ions (Q+Zn) and assessed its corresponding anticancer potential. In HepG2 cells, due to the known anticancer activity of several metal complexes [9,10,12,26], a novel quercetin–zinc (II) complex was evaluated. Conversely, HCT116 cells were also employed due to a limited understanding of metal complex interactions. To assess apoptosis induction and its underlying mechanism, we examined the caspase pathway activation via quercetin–zinc (II) complexes. We also sought to track the subcellular localization of these complexes using the fluorescent labeling of zinc ion, aiming to clarify the link between cellular uptake/localization of the complexes and apoptosis induction.

To our knowledge, no study has characterized the structure of the complex formed more spontaneously by the co-addition of quercetin and zinc ions in cultured cell media. Limited studies have also compared the functional differences between it and synthetic quercetin–zinc (II) complexes. Furthermore, this study revealed that the induction of apoptosis via quercetin–zinc (II) complexes was due to the activation of the caspase pathway, and that the marked increase in the cellular uptake of quercetin and zinc upon complexation may contribute to its anticancer activity.

## 2. Results and Discussion

### 2.1. Spectroscopic Characterization of Zn-Q and Q+Zn

#### 2.1.1. Analysis of Synthetic Zn-Q

Structural analysis of the novel synthesized Zn-Q was comprehensively performed by 1D ^1^H and 2D correlation spectroscopy (COSY), nuclear magnetic resonance (NMR), Fourier-transform infrared spectroscopy(FT-IR), ultraviolet–visible spectroscopy (UV-Vis), electrospray ionization mass spectrometer (ESI-MS), and X-ray diffraction analysis (XRD) analysis.

**Figure 1:**
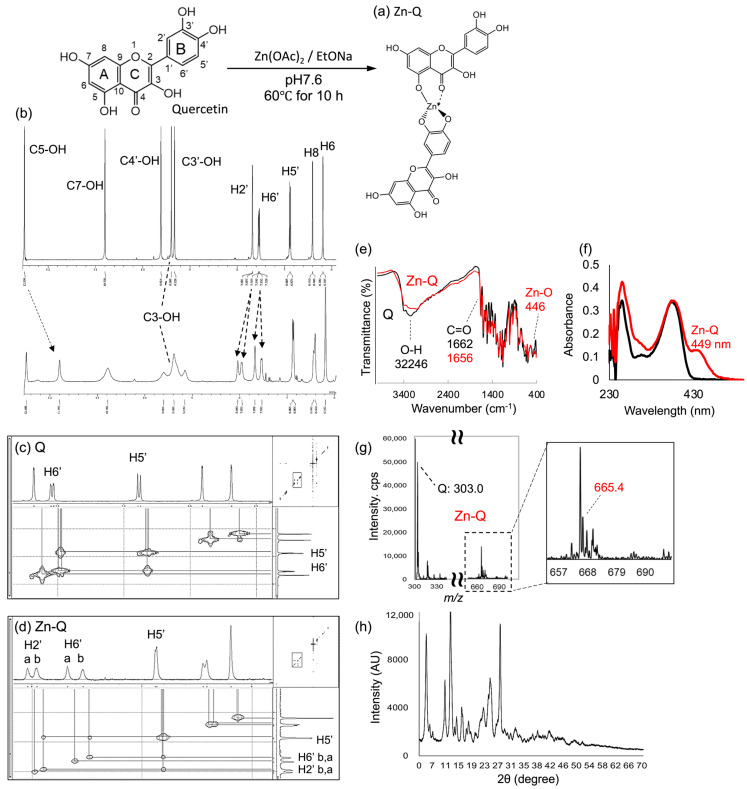
Synthetic scheme and structural characterization of Zn-Q (**a**) Presumed structures of Zn-Q. (**b**) The ^1^H-NMR spectra of quercetin (**upper**) and synthesized Zn-Q (**bottom**). 2D COSY-NMR spectra of quercetin (**c**) and Zn-Q (**d**). (**e**) FT-IR spectra of quercetin (Q; black) and Zn-Q (red) in the wavenumber range of 400–4000 cm^−1^. (**f**) UV-Vis spectra of quercetin (black) and Zn-Q (red) in the wavelength range of 230–550 nm (**g**) Electrospray ionization mass spectra of Zn-Q. (**h**) Powder XRD patterns of Zn-Q.

The ^1^H-NMR chemical shift assignments of quercetin were based on the report of Kyriakou et al. [27]. Figure 1b showed that upon complexation, all hydroxyl peaks except for H6 were broadened. The H2′ and H6′ proton peak split into two distinct peaks, suggesting that two quercetin molecules adopted different configurations. This was further supported by their integration ratios (Table A1). The intensities of the peaks corresponding to the C4′-, C3′-, and C5-OH groups diminished, along with a decrease in their integration ratios. Particularly, the peak for the C5-OH group, originally at 12.49 ppm, disappeared and shifted to 11.79 ppm. Considering that the peaks of each hydroxy group showed an integration ratio of 5-OH: 4′-OH: 3-OH: 3′-OH=1H: 1H: 2H: 1H, it was suggested that two molecules of quercetin had formed a complex with zinc (II). Specifically, one quercetin molecule likely binds to zinc (II) through the C4′- and C3′-OH groups, while the other engages via the 5-hydroxyl-4-carbonyl site. In agreement with Wei and Guo’s report [28], the protons (H2′, H5′, H6′) on the B ring showed broadening and a shift of more than 0.1 ppm, which indicates coordination at the chelating site of the B ring. On the other hand, the H6 and H8 peaks of Zn-Q broadened and shifted toward the low-field side, as in the zinc (II) complex of rutin [28]. This was another indication that zinc (II) was coordinately bound to the 5-hydroxyl-4-carbonyl moiety of quercetin. The 2D COSY-NMR spectral analysis was conducted to elucidate detailed zinc (II) binding site interactions. The coupling between the H′6 and H′5 protons was obtained from the COSY spectrum of quercetin (Figure 1c). The coupling in quercetin was consistent with that reported by Le Nest et al. [29]. In the 2D COSY NMR spectrum of Zn-Q, a diagonal peak between 9 ppm and 11 ppm was not observed (Figure S1). Conversely, as depicted in Figure 1d, new cross-peaks involving H′5 and H′2 protons, undetected in quercetin, were identified. Metal complexes often induce parameter relaxation, which may have weakened the NMR signal and consequently made the COSY spectrum difficult to detect. Lehr et al. observed not only the anticipated through-bond coupling cross-peaks but also through-space and exchange cross-peaks in COSY spectra through the analysis of Co (II) and Fe (II) mononuclear complexes, as well as Co4L6 cage-type complexes [30]. The phenomena observed in mononuclear complexes with multiple ligand environments and rapid ligand exchange suggest that the H′5 and H′2 proton bonds exhibited by Zn-Q may be spatial cross-peaks similar to those in these complexes.

The FT-IR spectrum of Zn-Q showed the new vibration signal at 446 cm^−1^ corresponding to the ZnO was presented in the quercetin–zinc complex but not in the quercetin spectrum (Figure 1e), as reported by Al-Gaashani et al. and Lee and Tuyet [13,31]. Valuable insights were derived from comparing the FT-IR spectra of quercetin and Zn-Q. The characteristic C=O stretching mode of quercetin, observed at 1662.2 cm^−1^, was shifted to 1656.5 cm^−1^ upon complex formation. This spectral shift indicates coordination of the quercetin’s carbonyl group to the metal ion, as corroborated by previous studies [13,19,32,33]. The ν(C-O-H) deformation mode observed at 1314 cm^−1^ in quercetin was shifted to 1346 cm^−1^ in addition to 1314 cm^−1^ in Zn-Q. Such a shift typically signifies an augmented bonding order, as often seen when metals interact with the ortho-phenolic ν(O-H) groups on the quercetin B ring (C4′- and C3′-OH groups).

In the UV-Vis spectrum, flavonoids exhibit two main absorption bands: 320–385 nm for the B ring (cinnamoyl, band I) and 240–280 nm for the A ring (benzoyl, band II). The UV-Vis absorption spectroscopy of the complexes is as represented in Figure 1f; three characteristics of absorption peaks for quercetin were seen at 260, 305, and 378 nm. Upon formation of Zn-Q complex, three absorption peaks were obtained at 260, 382, and 438 nm. In addition to the overall shift of the wavelength of Zn-Q toward longer wavelengths compared to free quercetin, a new peak at 438 nm was identified, possibly due to the new ring formation. This long wavelength shift with complex formation was consistent with the trend reported by Shastrala et al. and Lee et al. [13,18]. Following various measurements, we conducted an ESI-MS analysis. The protonated ligand, [M + H]+, appeared at *m*/*z* 665.4, and species corresponding to the formation of a 2:1 complex between quercetin and Zn (II) were detected (Figure 1g).

Powder XRD analysis was used to determine the unique phase of Zn-Q and provided information on its crystallinity as a material. The diffractogram of Zn-Q revealed robust peaks indicative of its physico-chemical properties, with 17 distinct reflections identified in the 2θ range from 3° to 70° (Figure 1h). Hazra et al. [34] and Papan et al. reported that the high crystal structure features of quercetin at diffraction angles 2θ values of 10°, 12°, 15°, 24°, 26°, and 27°. Apart from these peaks derived from quercetin, peaks specific to Zn-Q, such as 5°, 16°, 18°, and 22° were observed. The X-ray diffraction peaks of Zn-Q were different in both intensity and angle compared to the diffraction peaks of the quercetin–iron complex, the quercetin–magnesium complex [32], and the zinc–quercetin complex formed on the zinc-modified SBA-15 silica [35]. In the quercetin–magnesium complex, each metal coordinated to site I on the C ring and site III on the B ring of quercetin. In contrast, Zn-Q showed a diffraction pattern different from that of the magnesium complex, reinforcing our speculation that it is a new structure with zinc coordinated to site II on the C ring and site III on the B ring.

Combined analysis of ^1^H and 2D COSY-NMR, FT-IR, UV-Vis, and ESI-MS suggested the Zn-Q structure shown in Figure 1a. Elemental analysis of Zn-Q showed a discrepancy of 1.5% for C% and 0.19% for H% compared to the theoretical calculation (C30H17O14Zn). Furthermore, atomic absorption measurements of Zn% also showed a discrepancy of 1.6% from the calculated value (Table S1). This discrepancy can be attributed to the fact that the purity of Zn-Q after purification was approximately 97%. The Zn-Q structure we obtained resembles one of the quercetin–iron complexes synthesized by Papan et al [20]. Papan et al. synthesized quercetin–iron complexes with iron ions coordinated to 3′,4′-dihydroxy groups by reacting under pH 12 conditions. It has been reported that the range of chelation sites of flavonoids increases with pH [28,36]. Cornard and Merlin have demonstrated that under acidic conditions, the 3-hydroxyl-4-carbony (site I) and 5-hydroxyl-4-carbonyl (site II) moieties of quercetin are involved in metal coordination, whereas under alkaline conditions, additional chelation sites (site III) also participate. In our synthesis, the chelating sites were expanded using sodium ethoxide, a strong base. Further, 60 °C for 10 h seemed to promote binding at less reactive sites, i.e., site III. However, the reason for the preferential binding at sites II and III, over the more readily binding site I, remains unclear. De Souza et al. reported that, using density functional theory calculations, the rotated B ring polyphenol structure exhibits the best agreement with both experimental and theoretical ^1^H NMR chemical shift patterns [37,38]. To accurately reveal the structure of this bond, particularly considering the rotation of the B ring in solution, single crystal structure analysis is necessary.

#### 2.1.2. Analysis of Predicted Conformational Changes of Quercetin and Zinc Sulfate in Cultured Cell Media

**Figure 2:**
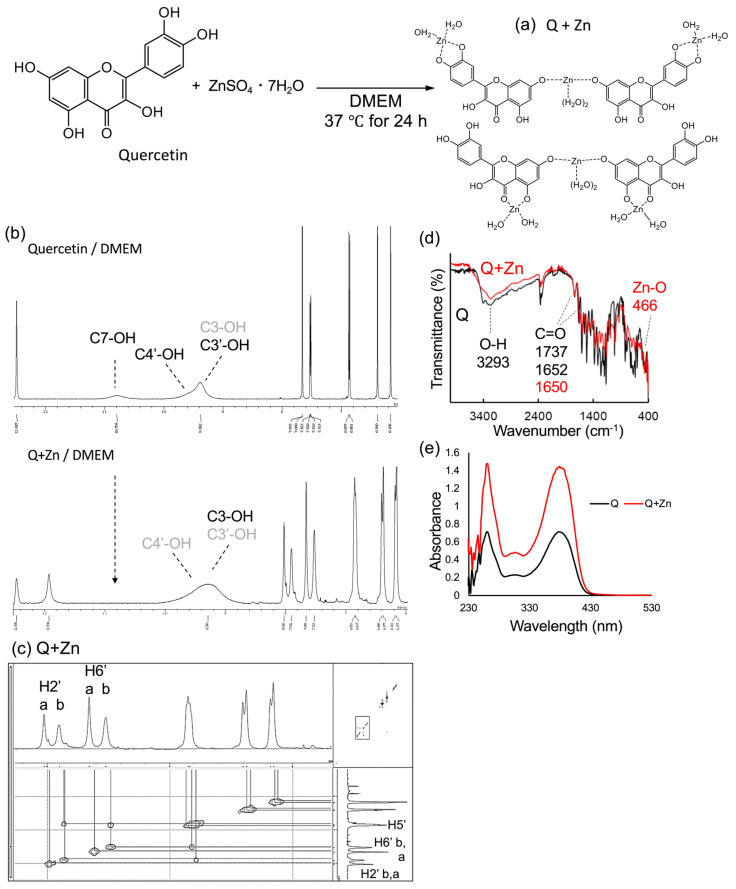
Analysis of the chelate structure of the Q+Zn complex in DMEM co-added with quercetin and ZnSO_4_ at a molar ratio of 2:1. (**a**) Estimated structure of Q+Zn complexes formed in DMEM. (**b**) The ^1^H-NMR spectra of quercetin (**upper**) and Q+Zn (**bottom**). (**c**) The 2D COSY-NMR spectra of Q+Zn. (**d**) FT-IR spectra of quercetin (Q; black) and Q+Zn (red) in the wavenumber range of 400–4000 cm^−1^. (**e**) UV-Vis spectra of quercetin (Q; black) and Q+Zn (red) in the wavelength range of 230–550 nm.

We subsequently assessed the conformational alterations in the medium upon co-treatment with quercetin and ZnSO_4_ in cultured cells using ^1^H-NMR and UV-Vis spectroscopy. As Figure 2b shows, when quercetin alone was incubated in Dulbecco’s Modified Eagle Medium (DMEM) for 24 h, all the C7-, C’4-, C3-, and C’3-OH peaks broadened. In particular, C’4-, C3-, C’3-OH merged into one peak. The broadening of the peak appeared to be due to chelation by trace minerals in the medium. The decrease in the integral ratio from 6H to 4H (Table A1), suggesting that the C3-OH peak was affected because the 3-hydroxy-4-carbonyl group (site I) is easily chelated by metal ions. Wei and Guo reported that the binding of zinc ions at site I of quercetin influences the 2′H and 6′H peaks [28]. In our spectral data, the 2′H and 6′H peaks were not broadened, suggesting that the chelating condition of Zn (II) ions at site I of quercetin in the cell culture medium might be a very loose coordination bond. In contrast, upon addition of zinc sulfate, two 2′H and two 6′H peaks were identified, respectively, and the C7-OH peak disappeared. The C5-OH peak was also identified at two different ppm values, although the integral ratio was less than 1H. Unlike the Zn-Q NMR peak, the broadening of the 2′H and 6′H peaks was mild, suggesting little coordination to C3-OH. Interestingly, COSY-NMR spectra showed the same interaction with Zn-Q (Figure 2c). Furthermore, this observation suggested that Q+Zn was indeed undergoing chelation with zinc ion.

The FT-IR spectrum of Q+Zn, similar to Zn-Q, revealed a new vibrational signal corresponding to ZnO at 466 cm^−1^ (Figure 2d). In contrast to the FT-IR spectrum of Zn-Q, Q+Zn exhibited an overall weaker vibrational signal compared to quercetin. Verification of the interaction between quercetin and the SiO_2_ matrix indicates a gradual broadening of absorption throughout the IR spectrum [39]. During iron complexation of quercetin, the same phenomenon was observed under deprotonated conditions. This observation implies that Q+Zn might be affected differently from Zn-Q by Zn (II) and compositional components in DMEM.

The UV-Vis spectral data in Figure 2e showed a 2-fold increase in the absorbance of Q+Zn, suggesting that quercetin was present in two equivalent amounts. Typically, metal coordination to quercetin’s sites I and III causes a bathochromic shift in bands I and II [40]. However, the absence of this shift suggests the co-treated chelate complex does not coordinate zinc (II) ions at these sites.

Based on these results, the structure may be bound to zinc (II) via C7-OH of quercetin and further chelated with zinc (II) ions at site I, as shown in Figure 2a. The deduced structure appears to be distinct from the quercetin–zinc (II) complexes in solution reported previously [41]. The structure of the zinc (II)-mediated 7-OH linkage of each of the two quercetin complexes agrees with the structure reported by Souza and Giovani for the rutin–zinc (II) complexes [40]. Powder XRD analysis of Q+Zn showed clear NaCl intermixing, with major diffraction peaks at 27°, 31°, 45°, 56°, 66°, and 75° (Figure S2). Peaks consistent with quercetin were identified at 22° and 27°, but the peak at 27° could be a mixture of quercetin and NaCl present in the DMEM. The mixture of Q+Zn and DMEM-derived components made it harder to determine the structural properties of the Q+Zn complex. Furthermore, significant deviations from the expected values were observed in the elemental analyses of CH% and Zn%. These deviations may also be attributable to the mixed composition of the medium (Table S1). In cell-based evaluation systems, the interaction of quercetin with serum and/or cellular metabolism may introduce additional complexity to the structural characterization of quercetin in the medium. Thus, the future structural analyses for Q+Zn in the medium should involve the purification of it from the medium.

### 2.2. Antioxidant Activity of Quercetin, Q+Zn, and Zn-Q

**Figure 3:**
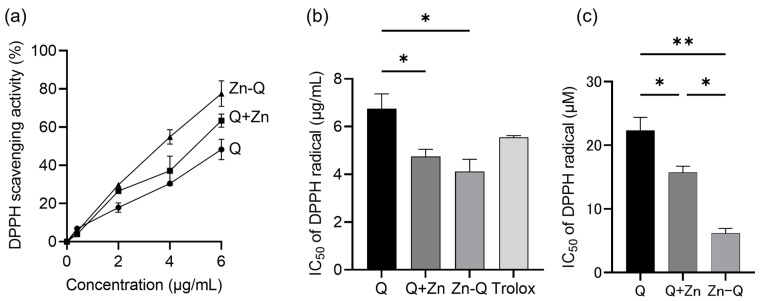
Antioxidant activity of quercetin (Q) and quercetin–zinc (II) complexes (Q+Zn or Zn-Q). (**a**) DPPH free radical scavenging activity rate and (**b**,**c**) IC_50_ value of Q, Q+Zn, or Zn-Q. Data from at least three independent triplicated experiments were presented as mean ± SD, *n* = 9. * mark denoted significant differences (* *p* < 0.05, ** *p* < 0.01) between Q and Q+Zn or Zn-Q, Q+Zn, and Zn-Q.

Quercetin’s various beneficial health effects, including its anticancer activity, arise in large part from its antioxidant properties. Metal coordination of quercetin leads to a significant increase in antioxidant capacity [25,32,42,43], although some metal complexes have been reported to cause the opposite effect [7,44]. Therefore, antioxidant capacity was evaluated via 2,2-diphenyl-1-picrylhydrazy (DPPH) radical scavenging assay. As depicted in Figure 3a, concentration-dependent radical scavenging was observed in the range of 0.4 to 6.0 µg/mL for quercetin, Q+Zn, Zn-Q, and Trolox. The IC_50_ values for DPPH radical scavenging were determined as 6.76 μg/mL (22.36 μM) for quercetin, 4.75 μg/mL (15.71 μM) for Q+Zn, 4.12 μg/mL (6.17 μM) for Zn-Q, and 5.55 μg/mL for Trolox (Figure 3b,c). Notably, Zn-Q exhibited a 3.6 times higher antioxidant capacity than quercetin and 1.4 times higher than Q+Zn. The higher antioxidant activity than the parent compound exhibited by Q+Zn was also found to be increased by chelation in the culture medium.

The major structural features required for antioxidant activity in flavonoids, which contribute significantly to their bioactivity, are the presence of site I, site II, and site III. Rodríguez-Arce and Saldías mention that the 3′ and 4′ ortho-dihydroxyl groups, i.e., site III, contribute most significantly to the antioxidant activity of flavonoids. Another report suggests that the antioxidant activity decreases when Sn (II) binds to both site I and site III [44]. Based on the structures suggested in Figure 1a and Figure 2a, within the Zn-Q complex, Zn (II) coordinates with one primary active site (site III) and another secondary active site (site II) of the two quercetin molecules. Conversely, in the Q+Zn complex, Zn (II) interacts with the active sites (site I) of both quercetin molecules and additionally occludes the 7-OH group. This distinct mode of coordination might be consequential in modulating the antioxidant activities of Zn-Q and Q+Zn.

### 2.3. The Antitumor Activity of Quercetin, Q+Zn, and Zn-Q

#### 2.3.1. Effect of Quercetin–Zinc (II) Complexes on the Inhibition of HepG2 and HCT116 Cells Proliferation

**Figure 4:**
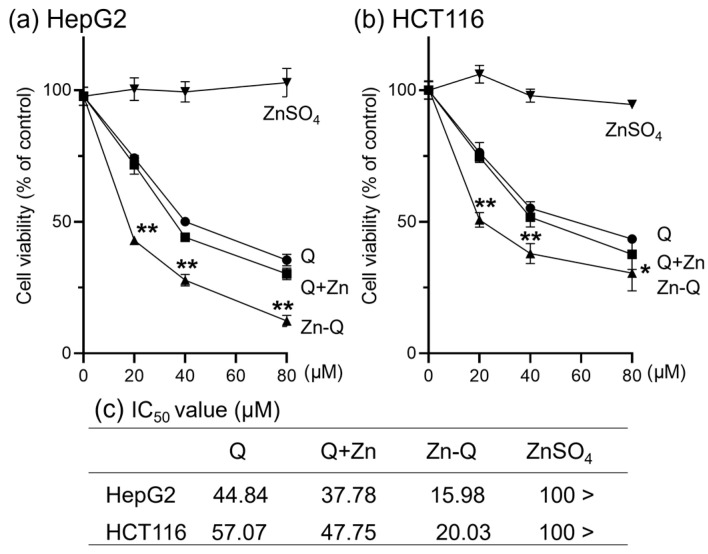
The viability of HepG2 and HCT116 cells treated with quercetin (Q), Q+Zn, or Zn-Q in a dose-dependent manner (**a**,**b**). (**c**) IC_50_ value of Q, Q+Zn, or Zn-Q. Both cell types were exposed to concentrations ranging from 0 to 80 μM of Q, Q+Zn, or Zn-Q for a duration of 48 h. Cell viability was colorimetrically assessed post-MTT staining and is represented as the optical density ratio of treated to control cells. The data are presented data with the mean ± SD, derived from three independent experiments and each performed in quadruplicate. * *p* < 0.05, ** *p* < 0.01 note significant differences between Q+Zn and Zn-Q.

We used the 3-(4,5-dimethylthiazol-2-yi)-2,5-diphenyltetrazolium bromide (MTT) assay to measure the basic ability of Q+Zn and Zn-Q to inhibit cancer cell proliferation. The quercetin and corresponding Zn (II) complexes were tested at concentrations ranging from 20 to 80 μM against HepG2 and HCT116 cell lines. As shown in Figure 4, in both HepG2 and HCT116 cells, Zn-Q showed a significantly stronger ability to inhibit cell proliferation than not only the parent compound quercetin but also Q+Zn. Quercetin and its co-addition with ZnSO_4_ (Q+Zn) also inhibited proliferation of both cells in a dose-dependent manner. Treatment with ZnSO_4_ alone had no effect on cell proliferation. The half-maximal inhibitory concentration (IC_50_) values shown in Figure 4c clearly indicated that Zn-Q had a 2.8-fold stronger inhibitory effect on cell proliferation in both cell types than quercetin. HepG2 cells were more sensitive to the antiproliferative effects of both of the quercetin–zinc (II) complexes than HCT116. The sensitivity was also observed for the parent compound, quercetin. Particularly in HepG2 cells, quercetin complexes have a stronger growth inhibitory effect than the ligand quercetin, consistent with the report by Tan et al. [9,10], suggesting that complex formation enhances the antiproliferative effect.

#### 2.3.2. Apoptosis Induction by Quercetin–Zinc (II) Complexes

**Figure 5:**
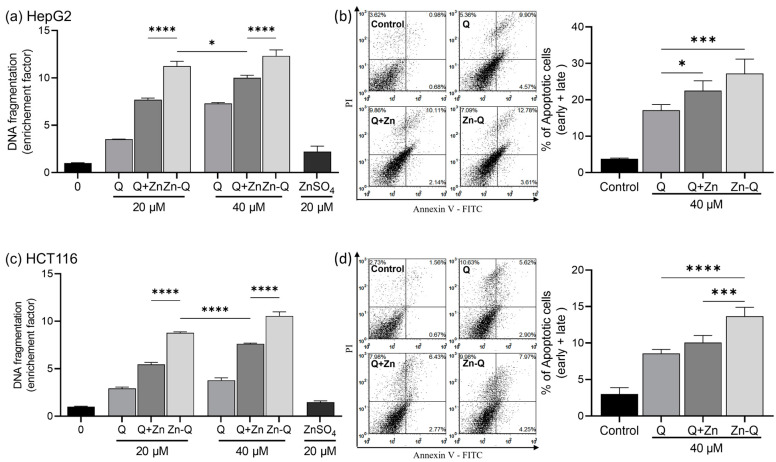
Apoptosis induction in HepG2 and HCT116 cells by Q, Q+Zn, or Zn-Q. (**a**,**c**) Detection of DNA fragmentation. Both types of cells were treated with 40 μM of Q, Q+Zn, or Zn-Q, or 20 μM of ZnSO_4_ for 48 h, followed by use of the Cell Death Detection ELISAPLUS Kit as described in Section 3. (**b**,**d**) Quantitation of % apoptotic fraction via flow cytometric analysis. Left panels indicate the representative flow histograms of Annexin V-FITC/PI fluorescence intensity and right graphs show the quantitative apoptotic (early + late) cell fractions. Both types of cells were treated with 40 μM of the indicated compounds for 48 h. * *p* < 0.05, *** *p* < 0.001, **** *p* < 0.0001 note significant differences between Q and Q+Zn or Zn-Q, Q+Zn, and Zn-Q.

The apoptosis-inducing effect of quercetin–zinc (II) complexes (Q+Zn and Zn-Q) on HepG2 and HCT116 cells was evaluated through the quantitation of histone-associated DNA fragment released into the cytosol or Annexin V/Propidium Iodide apoptosis detection. As shown in Figure 5a,c, quercetin, and its complexes with zinc (II), Q+Zn, and Zn-Q, markedly increased the release of histone-associated DNA fragments into the cytoplasm. This elevation, a hallmark of apoptosis, was observed in a concentration-dependent manner. The effect was especially pronounced in cells exposed to Zn-Q and remained consistent across both cell types. The ability to induce DNA fragmentation was more pronounced in Zn-Q > Q+Zn > Q sequences than in DMSO-treated controls. Interestingly, although the amount of quercetin in 20 μM Zn-Q was equivalent to that in 40 μM Q+Zn, the release of DNA fragmentation was notably higher in both HepG2 and HCT116 cells when treated with Zn-Q. Treatment with zinc sulfate alone did not induce apoptosis in HepG2 or HCT116 cells, indicating the combined effect of zinc (II)- and quercetin-enhanced apoptosis. Furthermore, apoptosis induced by the quercetin complex was determined by the annexin V/PI assay (Figure 5b,d), with outcomes aligning with the observed DNA fragmentation. Notably, the most potent Zn-Q treatment led to apoptosis in 27.2% of HepG2 cells and 13.6% of HCT116 cells after 48 h. This is in line with the data on cell proliferation inhibition (Figure 4), highlighting that HepG2 cells were more susceptible to apoptosis induction than HCT116 cells.

Consistent with our findings, the anticancer activity of flavonoid metal complexes is often reported to exceed that of their corresponding parent compounds. Lee et al. found that complexes of quercetin at site I with zinc (II) coordination led to a 1.4- to 2.0-fold increase in apoptosis compared to quercetin alone in DNA fragmentation assays [13]. Similarly, our results demonstrated enhanced apoptotic induction with the complexed quercetin. Notably, both our study and Lee et al.’s observed no apoptosis with Zn (II)-only treatments, underscoring the significance of complex formation in apoptosis induction. Additionally, when comparing Q+Zn and Zn-Q with equivalent quercetin content, Zn-Q exhibited a notably higher DNA fragmentation release (Figure 5a,c), suggesting that the complex’s specific structure also influences its pro-apoptotic potential.

#### 2.3.3. Zn-Q Significantly Induced Activation and Upregulation of Caspase-8, -9, and -3 Proteins

**Figure 6:**
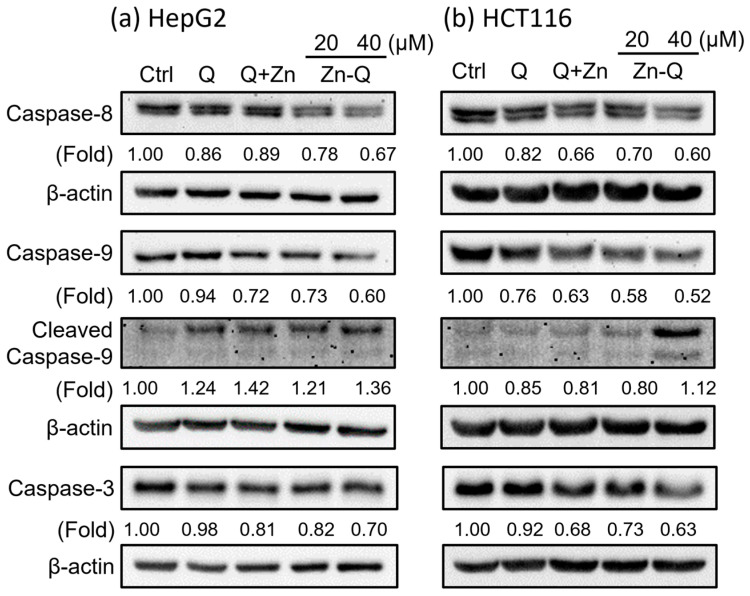
Zn-Q induced the activation of caspase-8, caspase-9, and caspase-3 in HepG2 cells and HCT116 cells. (**a**) Caspase-8, caspase-9, and caspase-3 in HepG2 cells. (**b**) Caspase-8 and caspase-9 in HCT116 cells. Cells were treated with quercetin (40 μM), quercetin (40 μM) plus 20 μM ZnSO_4_ (Q+Zn), or Zn-Q (20 μM or 40 μM) for 48 h, then the harvested cell lysate was used for western blot analysis. Ctrl, control group; Q, quercetin group; Q+Zn, quercetin and ZnSO_4_ co-treatment group; Zn-Q, zinc–quercetin complex group. Experiments were performed at least three times independently.

**Figure A1:**
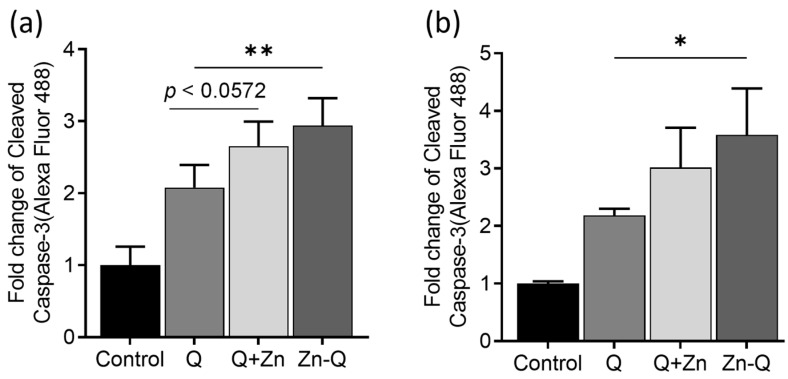
Cleaved form increased of caspase-3 in HepG2 and HCT116 cells after treatment with quercetin and Q+Zn and Zn-Q. Flow cytometric analysis of cleaved caspase-3 in (**a**) HepG2 and (**b**) HCT116 cells. Data from at least three independent triplicated experiments are presented as mean ± SD, and the * mark denotes significant differences (* p < 0.05, ** p < 0.01) between Q and Q+Zn or Zn-Q.

Given the caspase-3 activity in HepG2 cell apoptosis induced by the quercetin–manganese (II) complex [10], we examined to see if Zn-Q and Q+Zn modulate the caspase–protein pathway. Western blot analysis, as shown in Figure 6a, revealed that 40 μM Zn-Q significantly activated caspase-8, caspase-9, and caspase-3 in HepG2 cells, indicated by a decrease in their respective procaspases. Similarly, in HCT116 cells, the same caspases were activated by Zn-Q (Figure 6b). Specifically, the blot for caspase-9 showed a pronounced cleaved peptide, representing a decrease in procaspase. Caspase-3 activity was further confirmed through flow cytometric analysis (Figure A1). Compared to quercetin, Zn-Q exhibited a more pronounced caspase-3 activity, whereas Q+Zn showed moderate, yet higher, activity. Zn-Q displayed a notably stronger effect in activating caspases compared to treatments with 40 μM quercetin or a co-treatment of 40 μM quercetin and 20 μM ZnSO₄. Thus, Zn-Q appears to possess a heightened capability to activate apoptosis-related caspases.

Quercetin’s stimulation of apoptosis contributes to its anticancer properties [45]. At concentrations exceeding 40 μM, quercetin’s pro-oxidant effect becomes dominant [46], leading to increased ROS generation and oxidative stress in cancer cells. This can activate caspase-9 and caspase-3, inducing mitochondria-mediated apoptosis [46]. Moreover, quercetin’s activation of caspase-8 has been documented [47,48,49], implying it might also stimulate the death receptor-associated apoptosis pathway. Previous research highlights quercetin’s ability to activate caspase-9 and caspase-3 in HepG2 [50,51] and HCT 116 cells [52,53], generally at concentrations above 50 μM. A study reported that, while 100 μM ZnCl2 alone did not activate caspase-3, its combination with varying concentrations of quercetin did—suggesting a synergistic effect [54]. The activity of quercetin–metal complexes has been extensively explored. Significant caspase activation has been observed with quercetin–manganese (II) and –nickel (II) complexes in HepG2 cells [55], and the quercetin–copper (II) complex was found to up-regulate caspase-3 activity in A549 cells [56]. Additionally, the quercetin–oxovanadium (IV) complex intensified caspase-3/7 activation in MDA-MB231 cells compared to quercetin alone [57]. It is noteworthy to mention that our study was the first to elucidate the caspase activity induced by the quercetin–zinc (II) complex. Thus, the quercetin–metal ion combination, or their complex form, might be a strategic approach to amplify caspase activity in cancer treatments.

Our current findings indicate that, at 40 μM, Zn-Q prominently activated caspase-8, -9, and -3 in both HCT116 and HepG2 cells. Its apoptotic effect surpassed that of solo quercetin or its co-treatment with ZnSO_4_. This emphasizes that the synthesis of complexes of quercetin with zinc (II) enhances its caspase-activating potential in cancer cells, suggesting a promising direction for cancer control.

### 2.4. Intracellular Localization and Absorption of Q+Zn and Zn-Q

The antitumor activity of many compounds has been attributed to their interaction with DNA base pairs. The interaction of flavonoid metal complexes with DNA has also been extensively studied. However, most of these studies have been based on in vitro or in silico methodologies [9,58,59,60]. Here, we used a zinc ion fluorescent probe (ZnAF-2 DA), we determined the subcellular localization of the quercetin–zinc (II) complex, and we attempted to elucidate its incorporation into the cell nucleus.

**Figure 7:**
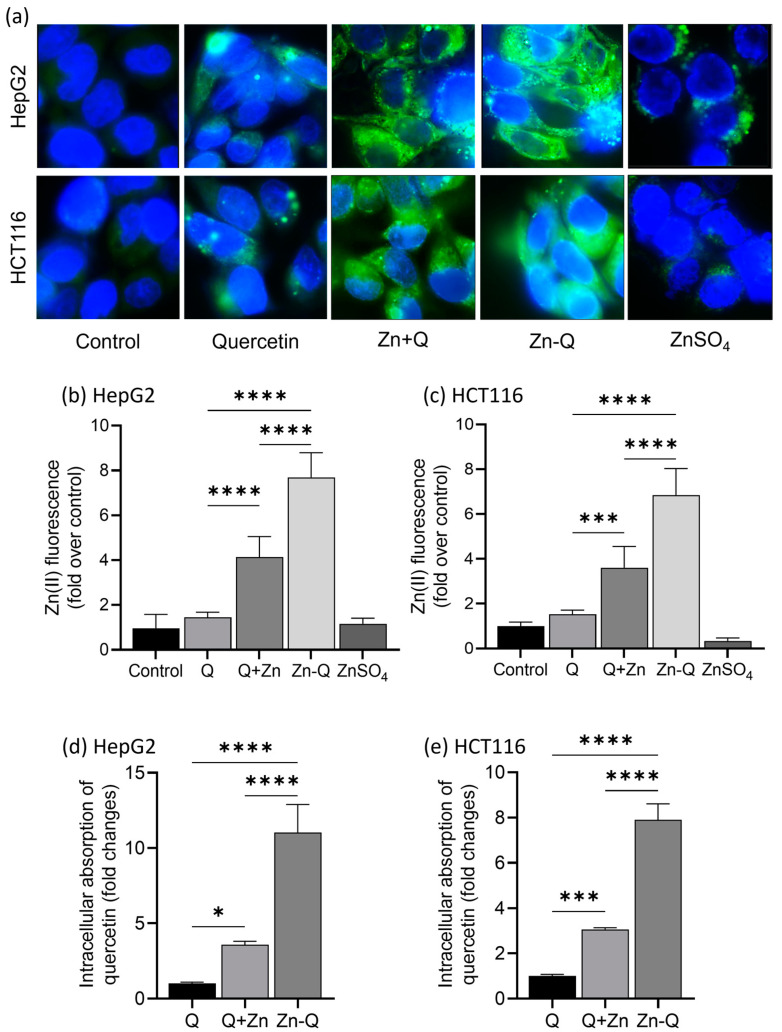
Intracellular localization and abundance of quercetin and quercetin–zinc (II) complexes. (**a**) Fluorescence microscopy images of ZnAF-2 DA-stained zinc ions in HepG2 (**upper panel**) and HCT116 (**lower panel**) after 3 h of culture. The microscope magnification was 60x and the scale bar indicates 10.0 μm. Quantitative analysis of ZnAF-2 DA-stained zinc ions in HepG2 (**b**) and in HCT116 (**c**). Quantitative determination of intracellular absorption of quercetin by HPLC analysis of HepG2 cells (**d**) and HCT116 cells (**e**). Data from at least three independent triplicated experiments are presented as mean ± SD, and * mark denotes significant differences (* p < 0.05, *** p < 0.001, **** p < 0.0001) between Q and Q+Zn or Zn-Q, Q+Zn, and Zn-Q).

Figure 7a clearly showed that zinc ion localizes more in the periphery of the nucleus in both HepG2 and HCT116 cells. The quercetin–zinc (II) complex-treated cells showed a significant intracellular presence of zinc ion. Using ImageJ1.50i software to quantify fluorescent images, we observed that the intracellular zinc concentrations were the highest following Zn-Q treatment. Specifically, concentrations were 5.28-fold higher than with quercetin in HepG2 cells and 4.48-fold higher in HCT116 cells. Furthermore, a significant differential absorption was also evident between Zn-Q and Q+Zn treatments (Figure 7b,c). Following Zn-Q, the Q+Zn treatment exhibited the next highest intracellular zinc uptake: an uptake 2.83-fold greater than quercetin in HepG2 cells and 2.36-fold greater than quercetin in HCT116 cells. Treatment with quercetin alone resulted in a modest increase in intracellular zinc ion levels, while adding only zinc sulfate maintained them at control levels. It is worth noting that this increase might be due to the enhanced uptake of zinc ions from the medium facilitated by quercetin.

We analyzed intracellular quercetin absorption using HPLC. Zn-Q treatment resulted in notable quercetin absorption increases of 11.0-fold in HepG2 cells and 7.9-fold in HCT116 cells, aligning with increased zinc absorption. Q+Zn treatment also significantly increased quercetin accumulation: 3.57-fold in HepG2 cells and 3.05-fold in HCT116 cells.

Fluorescence microscopy did not show notable zinc indicator localization in the nucleus, even with increased intracellular zinc ion from the quercetin–zinc (II) complex treatments. These findings do not exclude potential interactions between flavonoid metal complexes and nucleic acids. The current method did not determine if zinc retained its quercetin complex structure upon cell incorporation. Super-resolution microscopy would offer finer nuclear-focused localization. Further, analysis of the zinc ion and quercetin levels in the extracted nuclei might allow for a more direct assessment.

Several studies have shown that certain dietary flavonoids, especially quercetin, affect zinc uptake [61], transport [62], and homeostasis [63]. Quercetin has shown ionophore effects on zinc ions [64,65]. Dabbagh-Bazarbachi et al. found that a complex of quercetin and zinc enhances its ionophore activity and promotes intracellular transport of zinc in Hepa 1–6 cells and liposomes. The present results support the idea that the enhanced zinc uptake was due to the ionophore activity of quercetin. Importantly, the present study revealed that the cellular uptake of the ionophore compound quercetin was also significantly enhanced. Donadelli et al. found that an elevation in intracellular zinc ion concentration, mediated by zinc ionophores, led to caspase-independent apoptosis accompanied by AIF release [66]. Based on this, our quercetin–zinc (II) complex might induce apoptosis via two distinct mechanisms: caspase-independent due to the rise in zinc ion concentration and caspase-dependent resulting from increased quercetin levels.

Interestingly, while both Q+Zn and Zn-Q were quercetin–zinc (II) complexes, Zn-Q displayed significantly greater intracellular uptake. Clergeaud et al. reported that, in general, compounds with superior chelating abilities possess enhanced ionophore activity. This suggests that Zn-Q forms a more stable zinc chelate compared to Q+Zn. Its structure, potentially more membrane-permeable than Q+Zn, could account for the observed differences in ionophore activity. Quercetin–iron complexes have been reported to increase lipophilicity and influence membrane phase transitions in lipid bilayers [67]. Tarahovsky et al. proposed that flavonoid metal complexes penetrate lipid bilayers at hydrophobic sites and link adjacent layers through hydrophobic protein pores [68]. Such interactions could modulate the cellular uptake of quercetin–zinc (II) complexes. The impact of these complexes on lipid bilayer properties might affect membrane protein organization and the formation of functional units pivotal in cell signaling and metabolism. Future research should further elucidate the interactions between quercetin–zinc (II) complexes and lipid bilayers.

## 3. Materials and Methods

### 3.1. Reagents

Quercetin, and MTT reagent were purchased from Sigma (St. Louis, MO, USA). The antibodies against caspase-3 (#9662), caspase-8 (#9746), caspase-9 (#9502), and the HRP-conjugated anti-rabbit (#7074) and anti-mouse (#7076) secondary antibodies were from Cell Signaling Technology (Beverly, MA, USA). The antibody against β-actin (A5441) was from Sigma-Aldrich (St. Louis, MO, USA).

### 3.2. Synthesis of the Zinc (II)–Quercetin Complex (Zn-Q)

For the synthesis, we adapted and made some modifications to the method described by Papan et al [20]. Quercetin (0.60 mol) was dissolved in 50 mL of methanol in a 200 mL three-neck bottle. Sodium ethoxide (NaOEt) was prepared separately by dissolving sodium in ethanol to achieve a 1 M concentration. The quercetin solution was added to the 1 M sodium ethoxide solution (0.1% *v*/*v*) prepared, and the pH value was adjusted to 7.6. Subsequently, 0.3 mol of solid zinc acetate anhydride was added to the solution. The reaction mixture was stirred at 60 °C for 10 h. To quench the reaction, 50 mL of cold water was added and left to stand for 1 h. The mixture was then filtered by suction and the resulting residue was dried in a desiccator. The dried product was redissolved in cold methanol, filtered, and the filtrate was evaporated to obtain a dark orange powder purified quercetin–zinc (II) complex (yield 74.8%).

### 3.3. Structural Characterization of Zn-Q

The chemical structure of Zn-Q was determined by ^1^H- and 2D COSY-NMR, UV-Vis, FT-IR, ESI-MS, and XRD analysis as follows. ^1^H-NMR and COSY-NMR spectra were measured using a nuclear magnetic resonance spectrometer 600 MHz (JEOL-ECA600, JEOL, Tokyo, Japan). ^1^H-NMR measurements were performed in 8 scans (1 min) and COSY-NMR measurements were performed in 96 scans (11 h 12 min). All samples were dissolved in DMSO-d6 and measured (2–10 mg/0.7 mL). UV-Vis: The UV maximum absorption wavelength was measured by NanoDrop2000 (Thermo Fisher Scientific, MA, USA). Each sample was adjusted to a concentration of 0.5 μM with DMSO, and 2 μL of each sample was placed on a multichannel sample stand and measured. FT-IR: Infrared absorption wavelengths were measured via an FT-IR/IRT-3000 ATR-30-Z spectrophotometer (JASCO Corporation, Tokyo, Japan). Attenuated total reflection (ATR) was performed by placing a powder sample over the entire surface of the ATR crystals and then pressing the sample firmly against the prism while compressing it. The measuring range was 400–4000 cm^−1^. ESI-MS: The molecular weight of the Zn-Q was determined via ESI-MS 3200QTRAP (AB SCIEX, Tokyo, Japan). The concentrations of the quercetin and Zn-Q were adjusted to 1~10 ppm with acetonitrile. The mass spectrometer was monitored in the positive mode. The optimized detection parameters were as follows: scan type, electrospray ionization (ESI), ionization mode: positive mode, curtain gas: 20 psi, collision gas: high, ion spray voltage: 5.5 kV, temperature: 600 °C, ion source gas 1: 50 psi, ion source gas 2: 80 psi, interface heater: ON. X-ray powder diffraction patterns were obtained using a PANalytical X’Pert PRO MPD instrument (Malvern Panalytical, Almelo, Netherlands). The measurements were conducted in the range of 3 to 80 degrees with a voltage and current of 45 kV and 40 mA. The measurement conditions were divergent slit (DS) size [°] 0.1089, sample width [mm] 10.00, measurement temperature [°C] 25.00, target: Cu. Elemental analyses were conducted for Zn-Q and Q+Zn samples using a PerkinElmer 2400II elemental analyzer (Perkin-Elmer, MA, USA) to determine the percentages of carbon and hydrogen. The atomic absorption spectrometry, performed using an Agilent 55B AA (Agilent Technologies, Santa Clara, CA, USA), was specifically employed for the measurement of Zn%. 

### 3.4. Structural Characterization of Q+Zn

For measurement of Q+Zn, quercetin and ZnSO_4_ solution, each adjusted to 100 mM, were added to phenol red and serum-free DMEM, respectively, at a molar ratio of 2:1 (final concentration 1 mM) and incubated at 37 °C for 24 h. Measurements of quercetin in DMEM were also prepared under the same conditions as Q+Zn. The precipitated compounds were collected by centrifugation, the resulting powder was dried, and 2–10 mg of each was dissolved in 0.7 mL DMSO-d6 for ^1^H- or COSY-NMR measurements. For UV-Vis measurements, quercetin and Q+Zn were also dissolved in DMSO in equal volumes. FT-IR, XRD, and elemental analysis, including CHN% and Zn%, were performed under the same conditions employed in the structural characterization of Zn-Q (Section 3.2).

### 3.5. Cell Culture and Sample Treatment

The human hepatocellular carcinoma cell line, HepG2, was sourced from the RIKEN Cell Bank (RCB1648) (Ibaraki, Japan). The human colorectal carcinoma cell line, HCT116, was obtained from the American Type Culture Collection (Manassas, VA, USA). These cells were maintained in DMEM supplemented with 10% fetal bovine serum and 1% penicillin-streptomycin glutamine. Cultures were incubated at 37 °C under a 5% CO_2_ atmosphere. Cells were seeded with the specified number of cells for the experiment, and, after 24 h of incubation, treated with the indicated sample at the specified time and concentration.

Quercetin and the synthesized Zn-Q were each dissolved in DMSO to 100 mM stock solutions and subsequently frozen. ZnSO_4_·7H_2_O was prepared as a 100 mM solution in H_2_O and similarly frozen. For the Q+Zn treatment, ZnSO_4_ solution was added at half the molar concentration of quercetin solution, based on the treatment concentration of quercetin.

### 3.6. 2,2-diphenyl-1-picrylhydrazy (DPPH) Free Radical Scavenging Activity

Radical scavenging activity of quercetin, Q+Zn, and Zn-Q, which differ in the number and position of free hydroxy groups, was determined via the DPPH method, a slight modification of our previously reported method [69]. Samples of quercetin (302.24 mg/mL) and Zn-Q (664.4 mg/mL) were prepared in DMSO. For Q+Zn, ZnSO_4_ solution was added for half equivalence of the quercetin concentration. Since Zn-Q was marginally soluble in methanol, the solvents for all samples were unified to DMSO. For the DPPH assay, ten microliters of each sample were dispensed into a 96-well plate and combined with 190 microliters of 0.2 mM DPPH dissolved in ethanol, resulting in final concentrations of 0.4, 2.0, 4.0, and 6.0 μg /mL for each sample. A control blank containing a mixture of DMSO and DPPH, adjusted to match the DMSO concentration in the samples, was employed, with the free radical scavenging effect of DMSO subtracted. As positive controls, Trolox standards were prepared at concentrations of 0.05, 0.1, 0.15, 0.2, and 0.25 mg/mL in DMSO. The plates were placed in the dark at 25 °C for 30 min with mixing once every 10 min, and then the absorbance at 492 nm was measured using a microplate reader (Thermo Scientific Multiscan FC, Tokyo, Japan). The percentage scavenging rate of DPPH was calculated according to the formula: DPPH scavenging rate = (A0 − As)/A0 ×100% where A0 represents the absorption of the blank sample and *As* represents the absorption of each sample or other standards.

### 3.7. Cell Viability Assay

The cell survival rate was measured via an MTT assay. In brief, HCT116 (7.0 × 10^3^/well) cells and HepG2 (1.2 × 10^3^/well) cells were plated into each well of 96-well plates. The MTT solution is then added to each well and incubated for another 4 h, after which 100 μL of medium is withdrawn. A total of 100 μL of DMSO was added to each well to dissolve the formazan. The amount of formazan was determined by measuring absorbance at 540 nm in a plate reader (Multiskan™ FC, Thermo Scientific™, Waltham, MA, USA). Cell viability was expressed as the optical density ratio of the treatment to the control.

### 3.8. ELISA Detection of DNA Fragmentation

DNA fragmentation was assessed using the Cell Death Detection ELISAPLUS kit (Roche Diagnostics, Basel, Switzerland) as per the manufacturer’s guidelines. HepG2 cells (9.5 × 10^4^ cells/well) and HCT116 cells (2.1 × 10^4^ cells/well) were seeded in 12-well plates and subjected to sample treatments. Post-treatment, cells were lysed and the lysates were centrifuged at 200× *g* for 10 min. The resulting supernatants were transferred to an ELISA plate and incubated for 2 h with an immunoreagent buffer containing anti-histone biotin and anti-DNA POD. After subsequent washing steps, 100 µL of ABTS substrate buffer was added. Absorbance was measured at wavelengths of 405 nm and 490 nm using a Multiskan^TM^ FC microplate reader. The enrichment factor for apoptosis induction was determined by comparing the optical density ratios of treated samples to control samples.

### 3.9. Apoptosis Detection by Annexin V-FITC/Propidium Iodide Flow Cytometry

Apoptosis quantitation was performed using the FITC Annexin V Apoptosis Detection Kit I (BD Biosciences, San Diego, CA, USA) following the manufacturer’s instructions. In brief, HepG2 (2.4 × 10^5^/well) or HCT116 (1.96 × 10^5^/well) cells were plated into each well of 6-well plates. After treatment with 40 μM of samples for 48 h, both cell lines were suspended in 100 μL of binding buffer and then incubated with FITC-Annexin V and propidium iodide (PI) staining solution for 15 min. Flow cytometric analyses were subsequently conducted, with cells being assessed at FL1 (530 nm) and FL3 (630 nm) channels (CyFlow^®^, Sysmex Partec GmbH, Görlitz, Germany).

### 3.10. Western Blot Analysis

Western blot analysis was performed as described previously [70]. Briefly, the harvested cells were lysed in the RIPA buffer containing 50 mM Tris-HCl (pH 8.0), 150 mM NaCl, 1 mM EDTA, 1% Nonidet P-40, 0.25% Na-deoxycholate, 1 mM sodium fluoride, 1 mM sodium orthovanadate, 1 mM phenylmethylsulfhonyl fluoride, and proteinase inhibitor cocktail (Nacalai Tesque, Kyoto, Japan). Following blocking, membranes were probed with specific primary antibodies overnight at 4° C, then with corresponding HRP-conjugated secondary antibodies for 1 h at room temperature. Protein bands were visualized using the ECL system and quantified with LumiVision Analyzer140.exe (TAITEC Co., Saitama, Japan).

### 3.11. Flow Cytometric Measurement of Caspase-3 Activity

The activity of caspase-3 was assessed using human/mouse cleaved caspase-3 Alexa Fluor^®^ 488-conjugated antibody (R&D systems, Milpitas, CA, USA) following the manufacturer’s instructions. In brief, HepG2 (2.4 × 10^5^/well) and HCT116 cells (1.96 × 10^5^/well) were plated into each well of 6-well plates. After treatment with 40 μM of samples for 48 h, cells were fixed with 2% paraformaldehyde at 37 °C for 10 min. Then, cells were suspended in 90% methanol and permeated on ice for 30 min. Cells were resuspended in BSA buffer (0.5% BSA and 0.15% glycine in PBS) and blocked at room temperature for 10 min, then 2.5 μL of antibody were added and incubated in darkness at room temperature for 30 min. Finally, cells were resuspended with 200 μL of PBS. The cells were analyzed at FL1 (530 nm) with the flow cytometry (CyFlow^®^, Sysmex Partec GmbH, Görlitz, Germany).

### 3.12. Measurement of Intracellular Zinc Localization and Content by Fluorescent Staining

ZnAF^®^-2 DA indicator (Goryo Chemical, Hokkaido, Japan) was used for fluorescent staining, and the available zinc ion in each cell was measured by using a modified method of Husam Dabbagh-Bazarbachi et al. [65]. Cells were treated with 40 µM each sample for 3 h and were washed three times with HBSS, then ZnAF-2 DA adjusted to 2 μM was added, incubated at 37 °C for 1 h. Subsequently, cells were washed with HBSS and treated with DAPI (10 ng/mL) for 5 min at room temperature to stain the nucleus. Cells were washed twice with HBSS and examined under a KEYENCE fluorescence microscope (BZ-X810, KEYENCE, Oosaka, Japan) at 60 objective magnifications.

The images taken were further analyzed to quantify and compare intracellular zinc concentrations. Image analysis was performed using Image J 1.50i software (Wayne Rasband, USA). Overall fluorescence intensity and background values were derived from these images to confirm intracellular zinc ion availability among the different samples. Three cell-free regions were designated, from which we obtained their mean fluorescence intensities. We then determined the average the mean of background (BAv). This background average, BAv, was subtracted from the overall mean fluorescence intensity of the entire image, denoted as MM. Multiplying the resultant mean value by the corresponding area yielded a value for each sample. Finally, by averaging the values from this process, we obtained a representative value for the samples. The integrated density (IntDen) was computed as the following:

IntDen = Area × (MM − BAv)(1)

Area: Area of the selected image (not applicable for background).

MM: Subtract the mean of BG (BAv) from the mean of the entire image.

BAv: Average the mean of the background.

IntDen: Integrated density, calculated by combining the area and median fluorescence intensity.

### 3.13. Uptake of Quercetin into HepG2 and HCT116 Cells

Quercetin and uptake in HepG2 and HCT116 cells were assessed based on a modified method by Wong et al. [71]. Cells were treated with 80 µM of quercetin, Q+Zn (80 µM of quercetin and 40 µM ZnSO_4_), or Zn-Q in a serum-free medium for 3 h. Post-treatment, cells were thrice washed with PBS containing 0.2% BSA and once with PBS alone. Cells were then harvested in 50% methanol and stored at −80 °C for over 24 h. For extraction, samples underwent 10 min sonication, then were mixed with ice-cold acetone (double the volume of methanol). After 1 h at −20 °C, they were centrifuged at 17,000× *g* for 5 min. The supernatant was evaporated at 30 °C and stored at −20 °C until HPLC analysis. Cellular uptake of samples was assessed using an HPLC unit (LC−2000Plus series; JASCO Corporation) with a COSMOSIL(R) πNAP column (4.6 mm I.D. × 250 mm; Nacalai Tesque, Japan). The solvent comprised water (A) and acetonitrile (B) at a flow rate of 1.0 mL/min, transitioning from 100% B over 25 min to 20% B by 30 min, then reverting to the initial 80:20 A:B ratio for 10 min. A photodiode array (PDA) detector was used for scanning, accumulating spectral data for all peaks in the absorption wavelength range 200–700 nm and recording chromatograms at 280 and 320 nm, respectively.

### 3.14. Statistical Analysis

The statistical analysis of data was determined via one-way analysis of variance (ANOVA) followed by Tukey’s multiple comparison test, using GraphPad Prism 9.5.1 software (San Diego, CA, USA). Data are expressed as the mean ± standard deviation (SD). All experiments were conducted in biological triplicates (*n* = 3) with at least three individual replicates, and uptake and metabolism experiments generated at least three biological replicates. The significance level was set as *p*-values less than 0.05.

## 4. Conclusions

We characterized two distinct quercetin–zinc (II) complexes: a chemically synthesized form (Zn-Q) and a naturally formed form under conditions similar to the in vivo environment (Q+Zn). Our findings demonstrated that both complexes effectively induced apoptosis in HepG2 and HCT116 cells, likely driven by the cellular uptake of Zn (II) and quercetin. Importantly, complexation brought out a higher potential than that of quercetin alone, with Zn-Q exhibiting a markedly superior activity compared to Q+Zn. Further studies are required to understand the interaction of the quercetin–zinc (II) complex with the lipid bilayer and to assess the impact of pH fluctuations on the complex in vivo.

## Data Availability

Data are contained within the article and its Supplementary Information file.

## Supplementary Materials

The following supporting information can be downloaded at: https://www.mdpi.com/article/10.3390/ijms242417457/s1.

## Appendix A

**Table A1:** H-NMR data for quercetin and complexes (Zn-Q, Q+Zn).

^1^H	Quercetin	Zn-Q
C5-OH	12.50 (1H, s)	11.79 (1H, brs)
C7-OH	10.79 (1H, s)	10.78 (2H, brs)
C4′-OH	9.61 (1H, s)	9.60 (1H, brs)
C3-OH	9.39 (1H, s)	9.38 (2H, brs)
C3′-OH	9.32 (1H, s)	9.15 (1H, brs)
H-2′	7.68 (1H, d, J = 2.4 Hz)	8.04 (1H, brs)/7.93 (1H, brs)
H-6′	7.54 (1H, dd, J = 3.0/ 3.0 Hz)	7.67 (1H, brs)/7.53 (1H, brs)
H-5′	6.88 (1H, d, J = 12.6 Hz)	6.87 (2H, d, J = 12.6 Hz)
H-8	6.41 (1H, d, J = 2.4 Hz)	6.42 (2H, d, J = 19.8 Hz)
H-6	6.18 (1H, d, J = 3.0 Hz)	6.19 (2H, s)
**^1^H**	**Quercetin/DMEM**	**Q+Zn/DMEM**
C5-OH	12.49 (2H, s)	-
C7-OH	10.79 (1H, s)	-
C4′-OH	9.38 (2H, s)	-
C3-OH	-	9.29 (2H, S)
C3′-OH	9.38 (2H, s)	-
H-2′	7.66 (2H, d, J = 2.4 Hz)	8.02 (1H, brs)/7.90 (1H, brs)
H-6′	7.51 (2H, dd, J = 3.0/ 3.0 Hz)	7.66 (1H, brs)/7.52 (1H, brs)
H-5′	6.87 (2H, d, J = 12.6 Hz)	6.85 (2H, d, J = 8.4 Hz)
H-8	6.39 (2H, s)	6.41 (2H, d, J = 1.8 Hz)
H-6	6.17 (2H, s)	6.18 (2H, d, J = 1.8 Hz)
