# Supplementary material for: Apoptosis Induction in HepG2 and HCT116 Cells by a Novel Quercetin-Zinc (II) Complex: Enhanced Absorption of Quercetin and Zinc (II)
Source: Int J Mol Sci. 2023 Dec 14;24(24):17457. doi: 10.3390/ijms242417457 (PMC10743889; doi:10.3390/ijms242417457)
Supplement: Supplementary file 1 [file ijms-24-17457-s001.zip › ijms-2720176-supplementary.pdf]

Table S1. Elemental analysis data of Zn-Q and Q+Zn

Complex	C%		H%		Zn%		**molecular formula**	**purity**
	calc.	found	calc.	found	calc.	found	expected	
Zn-Q	54.0	52.5	2.60	2.79	9.8	8.2	$C_{30}H_{17}O_{14}Zn$	97%
Q+Zn	39.8	43.7	3.10	2.98	21.6	7.1	$C_{30}H_{28}O_{20}Zn_3$	ND

ND : Not Determined

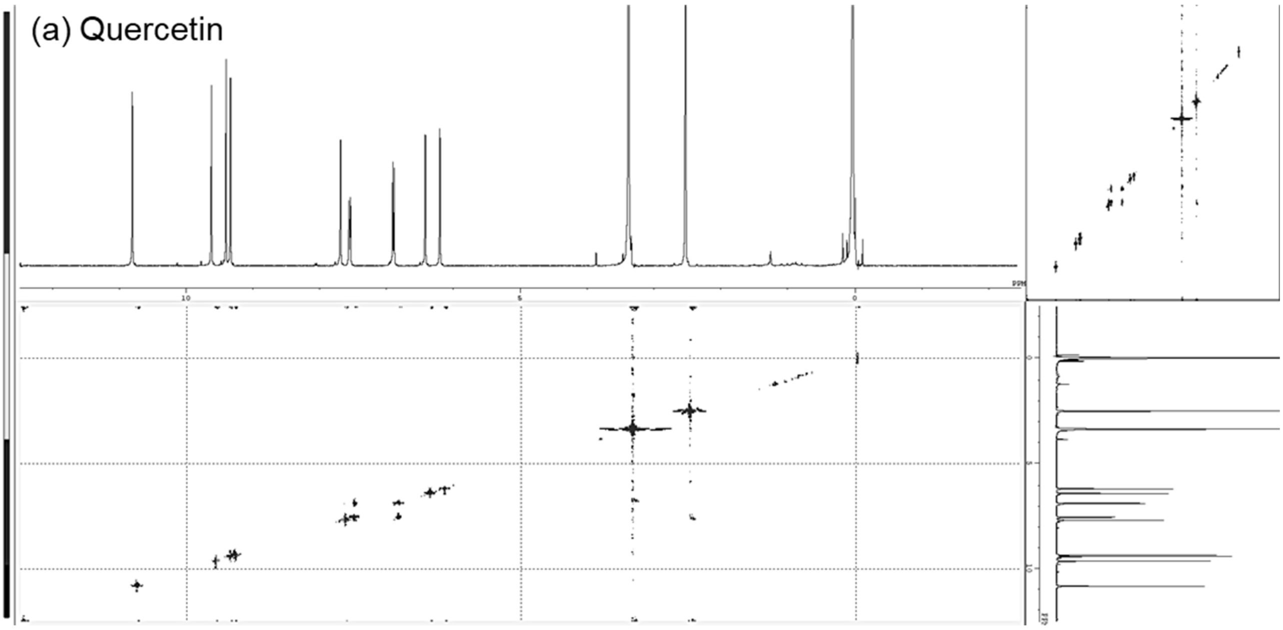

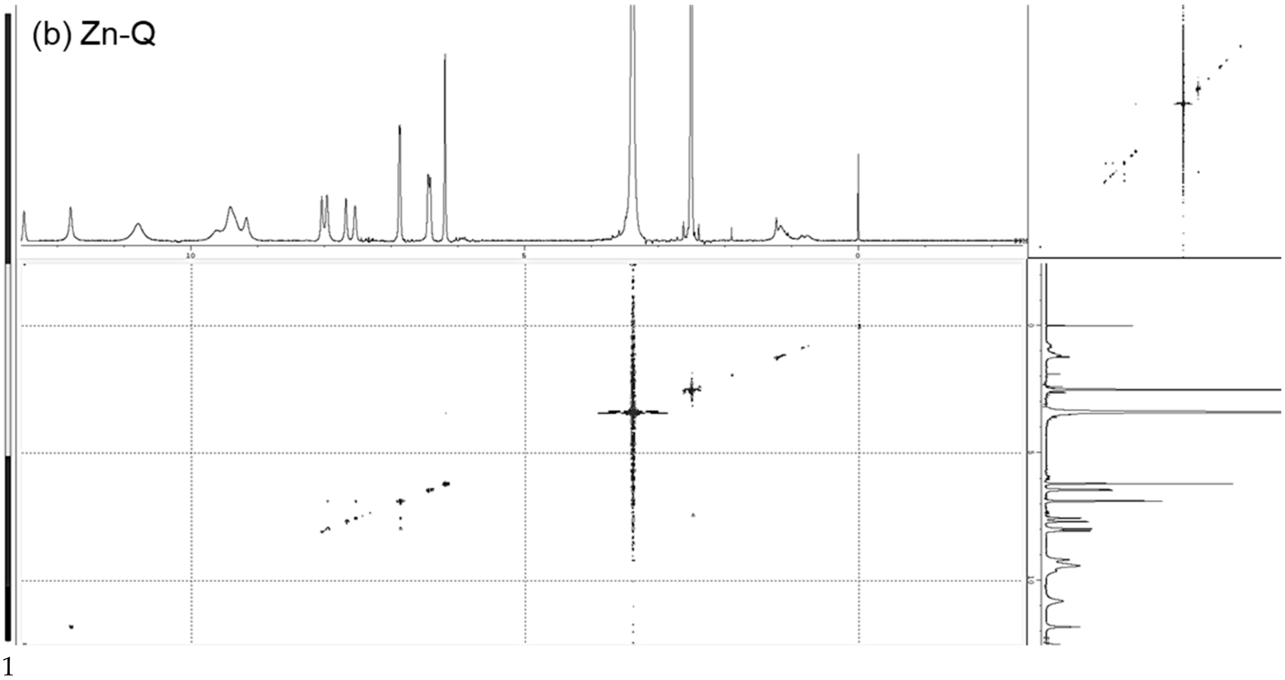

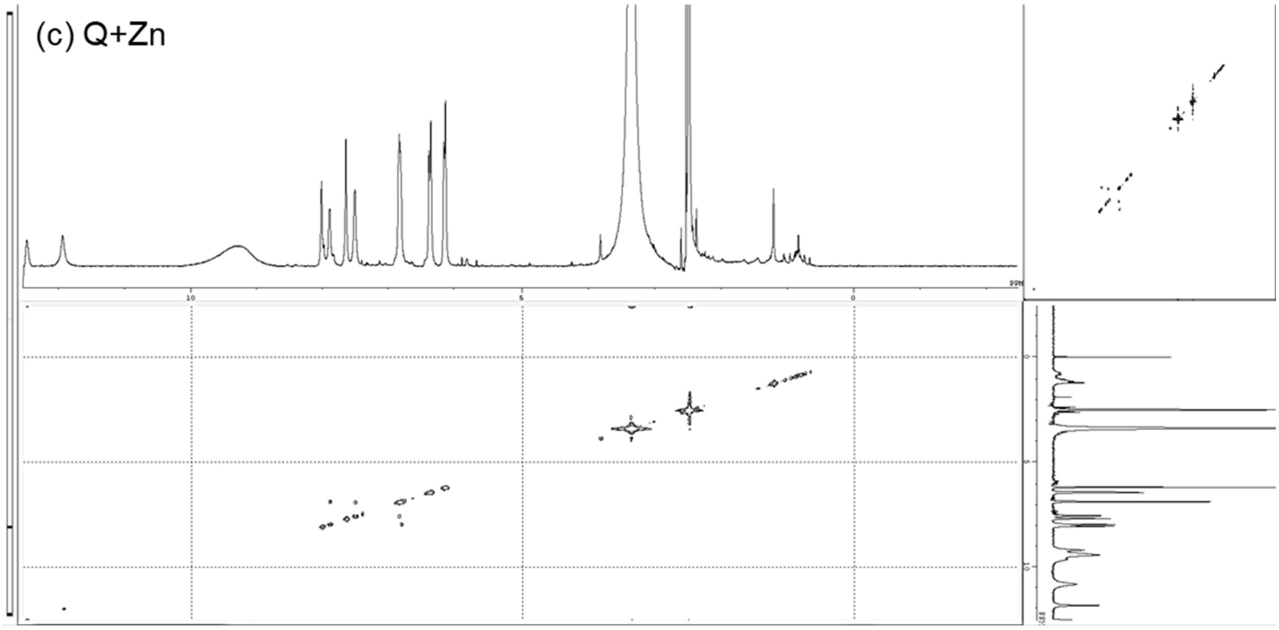

Figure S1: 2D COSY-NMR spectra of Quercetin (a), Zn-Q (b), and Q+Zn (c).

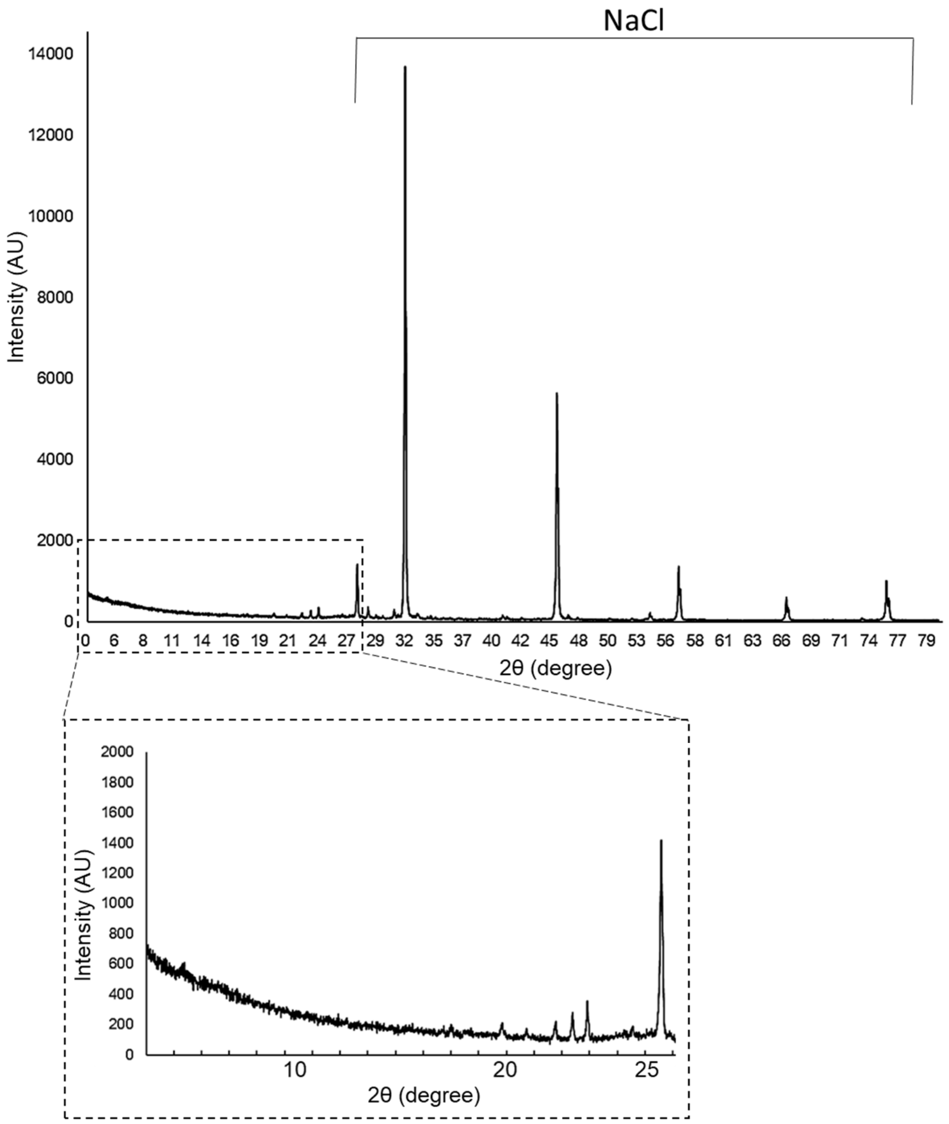

Figure S2: Powder XRD pattern of Q+Zn. The overall crystal structure (top) and the amorphous structure shown by the magnified image in the 2θ range 3° to 40° (bottom). The diffraction angles 2θ of 31°, 45°, 56°, 66°, and 75° indicated characteristic peaks of the NaCl crystal structure.
